# In this month’s *Bulletin*

**DOI:** 10.2471/BLT.16.000316

**Published:** 2016-03-01

In the editorial section, Christopher Dye et al. (158) encourage all researchers to share their data on Zika virus as quickly and widely as possible. Michelle J Hindin et al. (159) argue that pregnancies as well as birth rates should be tracked as development indicators. Yoko Akachi et al. (160) call for papers measuring quality of clinical care in low- and middle-income countries.

In the news section, Carolyn Mahoney and Fiona Fleck report on how surgical provision falls far short of what is needed in developing countries (163–164). Ana Bispo tells Andréia Azevedo Soares how scientists are scrambling to develop better tests for Zika infections (165–166). 

## Chile 

### School results and dietary habits

Paulina Correa-Burrows et al. (185–192) explore the associations between a healthy diet and academic performance among 16 year-old students.

## China

**Supporting elderly people with dementia **

Christina Wu et al. (167–173) evaluate health care and support in a rural area. 

**HIV-related services online **

Weibin Cheng et al. (222–227) describe a community-based project to expand access to testing and treatment services for HIV. 

## China, India, Malaysia, Republic of Korea, Singapore, Thailand and Viet Nam 

**Catastrophic medical bills **

Stephen Jan et al. (193–200) examine the out-of-pocket costs of hospitalization for acute coronary syndromes.

## Guinea, Liberia and Sierra Leone 

**Surviving Ebola **

Tine Van Bortel et al. (210–214) study the psychosocial impacts of the epidemic on affected individuals and communities. 

## Norway

### Estimating cancer incidence 

Sebastien Antoni et al. (174–184) compare GLOBOCAN estimates with national data. 

## Global 

### Provision of surgical services 

Thomas G Weiser et al. (201–209) estimate global surgical volumes and gaps in 2012.

### Household air pollution 

Adeladza Kofi Amegah & Jouni JK Jaakkola (215–221) recommend actions for improving indoor air quality.

### Producing the world’s food

Andrew D Jones & Gebisa Ejeta (228–229) propose changes to the way food crops are produced. 

### Tracking disability and death

Nicola C Richards et al. (230–232) argue that more data are needed on disabilities as outcomes of non-communicable diseases. 

